# Activation of ZBP1/RIPK3/MLKL-Dependent Necroptosis by Pseudorabies Virus Restricts Viral Infection in BV2 Microglia Cells

**DOI:** 10.1155/tbed/8510846

**Published:** 2025-10-07

**Authors:** Yiyu Liu, Haolin Li, Yu Dai, Jingyi Niu, Linhan Jiang, Jia Tang, Rendong Fang, Chao Ye

**Affiliations:** Joint International Research Laboratory of Animal Health and Animal Food Safety, College of Veterinary Medicine, Southwest University, Chongqing 400715, China

**Keywords:** mixed lineage kinase domain-like, necroptosis, pseudorabies virus, receptor-interacting protein kinase 3, *Z*-nucleic acid-binding protein 1

## Abstract

Pseudorabies (PR) virus (PRV), also known as suid herpesvirus 1, is an alphaherpesvirus that causes huge economic losses in the global swine industry. Necroptosis, a caspase-independent programmed cell death, has been demonstrated to play an important role in regulating viral infections. Here, we showed that PRV infection triggered receptor-interacting protein (RIP) kinase (RIPK) 3/mixed lineage kinasedomain-like (MLKL)-dependent necroptosis in BV2 microglial cells. Mechanistically, PRV infection induced the formation of *Z-DNA* and upregulated the expression of Z-nucleic acid-binding protein 1 (ZBP1), leading to ZBP1–RIPK3 interaction that triggered the RIPK3/MLKL-dependent necroptosis pathway. Additionally, both RNA interference and protein overexpression experiments demonstrated that RIPK3 and MLKL independently restricted PRV infection in BV2 microglial cells. The underlying mechanism lay in the fact that RIPK3 and MLKL can independently enhance the interferon (IFN)-β signaling pathway. Similarly, pharmacological inhibition of RIPK3 and MLKL using GW806742X and UH15-38 promoted PRV infection in BV2 microglial cells; however, this treatment did not affect IFN-β pathway activation. Taken together, our study elucidated the molecular mechanism of ZBP1/RIPK3/MLKL-dependent necroptosis activation during PRV infection and revealed that necroptosis signaling exerted anti-PRV effects in BV2 microglia cells. These findings provided novel insights into the antiviral defense mechanisms of microglia in the central nervous system (CNS) and suggested that targeting necroptosis signaling may offer a therapeutic strategy for controlling PRV infection.

## 1. Introduction

Pseudorabies (PR) virus (PRV), classified as suid herpesvirus 1, is the etiological agent responsible for PR, which results in substantial economic damage on swine production worldwide [1]. PR in swine is characterized by lethal encephalitis in piglets, severe respiratory signs in adult pigs, and reproductive failure in pregnant sows [2]. Although pig is the only natural host for PRV, PRV also infects nonporcine domestic and wild hosts and usually causes severe itching and acute death [3]. Emerging evidences indicate that PRV can also infect humans and cause viral encephalitis [4–6], which raises concerns regarding the public health implications of PRV infection.

Programmed cell death (PCD) is an autonomous form of cell death that governs by a series of genes [7], and it is an important immune mechanism for the host to defend against bacterial and viral infection [8]. Apoptosis, pyroptosis, and necroptosis are the most well-defined PCD pathways, which are intricately involved in both homeostasis and disease [9]. Apoptosis, the first described PCD, plays important roles in eliminating virus-infected cells and maintaining homeostasis. Studies have found that PRV infection can induce host cell apoptosis in vitro and in vivo [10–12], suggesting the significance of apoptosis in replication and pathogenicity of PRV. A recent study further demonstrated that gM (a late protein encoded by PRV *UL10*) induced mitochondria-dependent apoptosis and enhanced the viral replication and pathogenicity [13]. Meanwhile, PRV infection was found to trigger pyroptosis via activation of the NLRP3 and IFI16 inflammasomes [14]. Furthermore, PRV UL4 protein was demonstrated to manipulate the inflammasome signaling and pyroptosis to enhance the pathogenicity of PRV [15]. Apart from apoptosis and pyroptosis, necroptosis has been found to be induced by PRV infection in PK-15 cells [16]; however, how PRV initiates necroptosis and how this process influences host innate immunity and viral infection remain unclear.

Necroptosis, a caspase-independent cell death pathway, has been considered as a host defense mechanism against viral infection [17]. Necroptosis in virus-infected cells has been found to be initiated by *Z*-nucleic acid-binding protein 1 (ZBP1), which senses and binds to nucleic acids in the *Z*-conformation (such as *Z*-DNA and *Z*-RNA) and then recruits and activates receptor-interacting protein (RIP) kinase (RIPK)3 [18, 19]. Upon activation, RIPK3 phosphorylates mixed lineage kinase domain-like (MLKL) and interacts with MLKL to form the necrosome complex. Phosphorylated MLKL in necrosome forms oligomers to drive plasma membrane leakage as the executioner of necroptosis [20, 21]. Recently, independent studies indicated that diverse viruses including RNA viruses (e.g., influenza A virus (IAV) [22] and severe acute respiratory syndrome coronavirus 2 (SARS-CoV-2) [23]) and DNA viruses (e.g., herpes simplex virus 1 (HSV-1) [24], hepatitis B virus [25], and porcine parvovirus [26]) activated ZBP1 to trigger necroptosis in infected cells. However, the precise function of ZBP1 during PRV-induced necroptosis remains to be clarified.

In this study, we found that PRV infection induced RIPK3/MLKL-dependent necroptosis in BV2 murine microglial cells. Mechanistically, PRV-generated *Z*-DNA activated the ZBP1/RIPK3/MLKL axis to trigger necroptosis activation. Notably, both RIPK3 and MLKL individually exerted anti-PRV effects by enhancing type I interferon (IFN-I) pathway activation in microglial cells. Pharmacological inhibition of RIPK3 or MLKL kinase activity also significantly reduced PRV infection in microglial cells. Taken together, our findings elucidated the molecular mechanism by which PRV activated ZBP1/RIPK3/MLKL-mediated necroptosis, highlighting this pathway as potential targets for the development of novel therapeutic strategies against PRV.

## 2. Materials and Methods

### 2.1. Cells and Virus

Mouse microglia (BV2), baby hamster kidney-21 (BHK-21), human embryonic kidney (HEK)-293T, and African green monkey kidney (Vero) cells were maintained in DMEM containing 10% fetal bovine serum (FBS) and antibiotics (100 U penicillin/mL and 100 μg streptomycin/mL). All cell cultures were maintained at 37°C in a humidified incubator containing 5% CO_2_.

PRV JS-2012 strain, a PRV variant, was maintained in our laboratory and stored at − 80 °C. Vero cells were used for both the preparation of viral stocks and the determination of infectivity titers. Viral titers were quantified using the 50% tissue culture infective dose (TCID_50_) assay, with calculations performed according to the Reed–Muench method.

### 2.2. Antibodies

The primary antibodies were shown as follows: anti-PRV glycoprotein E (gE) antibody (Ab) (preserved in our lab), anti-*p*-RIPK3 Ab (ab222320, Abcam, UK), anti-RIPK3 Ab (2283, ProSci, USA), anti-*p*-MLKL Ab ([ET1705-51, HUABIO, China] and [ab196436, Abcam, UK]), anti-MLKL Ab (ET1601-25, HUABIO, China), anti-ZBP1 Ab (Zippy-1) (AG-20B-0010-C100, AdipoGen, USA), anti-*Z*-DNA/*Z*-RNA (Z22) (Ab00783-23.0, Absolute, UK), anti-β-actin Ab (AA128, Beyotime, China), mouse anti-FLAG tag Ab (8146, Cell Signaling Technology, USA), rabbit anti-FLAG tag Ab (HY-P87166, Sigma Aldrich, USA), mouse anti-HA tag Ab (66006-2-Ig, Proteintech, China), and rabbit anti-HA tag Ab (HY-P80948B, Sigma Aldrich, USA). The second antibodies used in this study were HRP-labeled goat anti-mouse lgG (*H* + *L*) (A0216, Beyotime, China), HRP-labeled goat anti-rabbit lgG (*H* + *L*) (A0208, Beyotime, China), goat anti-mouse IgG (H&L) Alexa Fluor 488 (ab150113, Abcam, UK), and goat anti-rabbit IgG (H&L) Alexa Fluor 594 (ab150080, Abcam, UK).

### 2.3. One-Step Growth Curve

The BHK-21 and BV2 *mic*roglial cells were plated in T25 culture flasks and incubated for 12 h prior to infection. Following this, cells were exposed to PRV JS-2012 (MOI = 1) for 1 h to permit viral adsorption. After adsorption, the viral inoculum was discarded, and the cells were washed thrice with DMEM containing 2% FBS. Fresh medium was added, and the infected cells were further incubated at 37 °C under 5% CO_2_. Supernatants were collected at indicated time points (3, 6, 9, 12, 24, 36, and 48 h postinfection) for viral titration. Viral titers in harvested supernatants were determined on Vero cells using the TCID_50_ assay and calculated by the Reed–Muench method. The one-step growth curves of PRV in BHK-21 and BV2 cells were generated and analyzed using GraphPad Prism.

### 2.4. Cell Viability Assay

Cell viability was evaluated with a Cell Counting Kit-8 (CCK-8) assay (Zeheng Biotech, China) following the manufacturer's instructions. For inhibitors treatment experiments, cells were plated in 96-well plates at a density of 1 × 10^4^ cells per well for 12 h. Then, cells were inoculated with the corresponding inhibitor for 1 h and then infected with PRV for 24 h. For ZBP1 knockdown and overexpression studies, cells were transfected with the corresponding siRNAs or plasmids in 12-well plates for the indicated time and then seeded into 96-well plates at 1 × 10^4^ cells/well. After 12 h of attachment, the cells were infected with PRV (MOI = 5) for 24 h. Finally, 10% CCK-8 reagent in fresh DMEM was inoculated into each well, and the plates were maintained in the dark for 1 h. Absorbance was then measured at 450 nm with a microplate reader (Bio-Rad, Japan). Cell viability relative to the uninfected/infected controls was determined and plotted. Cell viability was determined using the following formula: viability = OD_450(sample)_/OD_450(control)_.

### 2.5. Inhibitor Treatment Assay

The apoptosis inhibitor, *z*-VAD-FMK (C1202), was purchased from Beyotime Biotech (Shanghai, China); the pyroptosis inhibitor necrosulfonamide (NSA) (HY-100573), the mouse MLKL inhibitor GW806742X (HY-112292), and the mouse RIPK3 inhibitor UH15-38 (HY-158312) were purchased from Med Chem Express (USA). All inhibitors were prepared according to the manufacturer's instructions prior to use. For inhibitors treatment experiments, the cells were pretreated with the corresponding compounds for 1 h, followed by PRV infection for 24 h.

### 2.6. Total and Soluble/Insoluble Protein Extraction

BV2 microglial cells were plated in 12-well plates at a density of 5 × 10^5^ cells per well prior to subsequent experimental treatments. For the extraction of total cellular protein, both floating and adherent cells were collected and lysed in 1 × SDS loading buffer (Beyotime, China) containing PMSF protease inhibitor (Beyotime, China) and PhosSTOP phosphatase inhibitor (Roche, USA). The resulting lysates were boiled for 5 min and stored at − 80 °C until further use.

Separation and extraction of soluble and insoluble fractions were carried out according to a previous study [27]. Briefly, both floating and adherent cells were collected and lysed in NP-40 lysis buffer. Then, the lysates were rotated for 4 h at 4 °C and centrifuged for 20 min (16,000 × *g*, 4°C). Supernatants were recovered and served as the soluble fractions. The cell pellet was washed with NP-40 lysis buffer and centrifuged for 10 min (16,000 × *g*, 4°C). The resulting pellet was then resuspended in 1 × SDS loading buffer (Beyotime, China) and subjected to sonication. After sonication, samples were boiled for 5 min and stored at − 80 °C until further use.

### 2.7. Western Blot Analysis

Cells were cultured in 12-well plates and subjected to treatments including PRV infection, siRNA, plasmids, or the corresponding inhibitors treatments. Following each treatment, the cells were lysed using 1 × SDS loading buffer containing PMSF protease inhibitor and PhosSTOP phosphatase inhibitor. The resulting lysates were separated by 12% SDS-PAGE and subsequently transferred onto a polyvinylidene difluoride (PVDF) membrane. After blocking with 5% nonfat milk or 5% BSA in 0.1% TBST buffer for 2 h at room temperature, the membranes were then incubated with primary antibody overnight at 4°C. After washing, the membranes were probed with an HRP-conjugated secondary antibody for 1 h at room temperature. Protein bands were visualized by enhanced chemiluminescence (ECL) reagents (Biosharp, China), and their intensities were quantified using ImageJ software.

### 2.8. Immunofluorescence Assay (IFA)

BV2 microglial cells were plated in 48-well plates (5 × 10^4^ cells per well) or confocal dishes (1 × 10^6^ cells per dish) (Biosharp, China) and cultured for 12 h before each treatment. Following the indicated experimental treatments, cells were fixed with ice-cold 4% paraformaldehyde (Sango Biotech, China) for 30 min, then permeabilized with 0.1% Triton-X 100 in PBS for 5 min, and blocked with 5% BSA in PBS buffer for 1 h at room temperature. After PBS washing, cells were inoculated with the indicated primary antibodies at 4°C overnight. Subsequently, goat anti-mouse IgG (H&L) Alexa Fluor 488 (Abcam, UK) or goat anti-rabbit IgG (H&L) Alexa Fluor 594 (Abcam, UK) was added at room temperature for 1 h. Following nuclear staining with DAPI (Beyotime, China) for 5 min, samples were mounted with antifading mounting medium (Solarbio, Beijing, China). Finally, the fluorescence images were visualized using either an inverted fluorescence microscope (Olympus, Japan) or an Olympus FV3000 confocal microscope (Olympus, Japan).

### 2.9. Quantitative Reverse Transcription-PCR (qRT-PCR)

The extraction of total RNA from the cultured cells was conducted using TRIzol reagent (Invitrogen, USA) according to the manufacturer's instructions. The cDNA was synthesized from the extracted RNA by PrimeScript FAST RT reagent Kit with gDNA Eraser (Takara, Japan). The mRNA levels of target genes were determined by qRT-PCR using the gene-specific primers (Table 1). Reactions were performed in a CFX Connect real-time PCR detection system (Bio-Rad, Japan) with 2 × SYBR Green Pro Taq HS Premix (AG11701, AG Bio, China). The relative mRNA expression was normalized to *β-actin* expression and analyzed via the 2^−ΔΔCT^ method. Additionally, absolute quantification of PRV gE transcript copies was performed using Premix Ex Taq (Probe qPCR, Takara) with an in-house assay [28]. The following primer and probe sequences were used: PRV-gE-forward: 5′–GAGTTCAGCAGCGACGAG–3′; PRV-gE-reverse: 5′–CGCCATAGTTGGGTCCATT–3′, and PRV-gE probe: FAM-5′–CGTCACTTCCGGTTTCTCCGGATC–3′-BHQ1.

### 2.10. Plasmids Construction and Transfection

The primer sequences for amplifying ZBP1 (GenBank: NM_021394.2), RIPK3 (GenBank: NM_001164108.1), and MLKL (GenBank: NM_001310613.1) were synthesized by Sangon Biotech (Shanghai, China) (Table 2). Subsequently, the open reading frames (ORFs) of target genes were amplified by PCR using cDNA from BV2 microglial cells as the template. The resulting PCR products were then cloned into either the pCAGGS or FLAG-tagged pCAGGS mammalian expression vector using a homologous recombination-based method. The recombinant plasmids were transfected into the corresponding cells using Lipofectamine 3000 Transfection Reagent (Invitrogen, USA) or Lipofectamine LTX Reagent (Invitrogen, USA), following the manufacturer's instructions. Then, cells were incubated for the indicated time prior to subsequent experiments.

### 2.11. RNA Interference

The sequences of siRNAs were synthesized by Sangon Biotech (Shanghai, China) and are shown in Table 3. Briefly, the *BV2* microglial cells were seeded onto 12-well plates prior to siRNA transfection. Then, cells were transfected with either control siRNA (si-Con) or the specific siRNAs at a final concentration of 50 nM, using Lipofectamine 3000 Transfection Reagent (Invitrogen, USA) in accordance with the manufacturer's guidelines. At 36 h after transfection, cells were stimulated with PRV for 24 h prior to subsequent qRT-PCR, western blot, and CCK-8 detection.

### 2.12. Co-Immunoprecipitation (Co-IP) Assay

HEK-293T cells were seeded into 6-well plates and transfected with transfection complex containing the corresponding plasmids for 24 h. Then, cells were lysed in ice-cold cell lysis buffer for western and IP (Beyotime, China). Following 30 min of cell lysis on ice, lysates were centrifuged for 10 min (12,000 rpm, 4 °C). The supernatants were split into input and IP fractions. For input fraction, supernatant was mixed with 1 × SDS loading buffer (Beyotime, China) and boiled for 5 min. For IP fraction, supernatant was incubated overnight at 4°C with target-specific antibodies and species-matched IgG controls. Antibody complexes were captured by incubation with prewashed Protein A/G Agarose Fast Flow beads (Beyotime, China) for 4 h at 4 °C with rotation and then centrifuged for 5 min (2000 rpm, 4°C). Finally, the beads were washed five times with PBS, resuspended in 1 × SDS loading buffer (Beyotime, China), and boiled for 5 min. All samples were stored at − 80 °C until further analysis. The precipitated proteins were analyzed by western blot using target-specified antibodies.

### 2.13. Statistical Analysis

Data were expressed as mean ± standard deviation (SD). Statistical significance between groups was analyzed with a two-tailed unpaired Student's *t*-test. Statistical significance was defined as follows: *⁣*^*∗*^*p*  < 0.05, *⁣*^*∗∗*^*p*  < 0.01, *⁣*^*∗∗∗*^*p*  < 0.001, *⁣*^*∗∗∗∗*^*p*  < 0.0001, and ns, no significance.

## 3. Results

### 3.1. PRV Infection Induced RIPK3/MLKL-Dependent Necroptosis In Vitro

**Figure 1:**
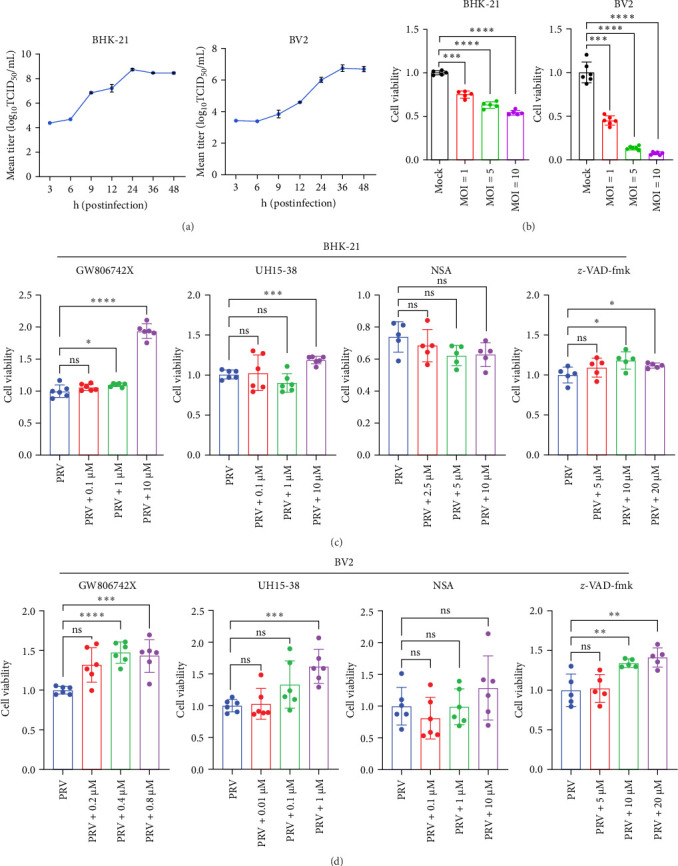
PRV infection induced RIPK3/MLKL-dependent cell death in BHK-21 and BV2 microglial cells. (A) The one-step growth curves of PRV in BHK-21 and BV2 microglial cells. (B) Cell viability of PRV-infected BHK-21 and BV2 microglial cells under different MOIs was assessed at 24 h postinfection. (C and D) Cell viability of PRV-infected BHK-21 and BV2 microglial cells following exposure to various inhibitors. Cells were pretreated with inhibitors for 1 h and then infected with PRV (MOI = 5) for 24 h. GW806742X, MLKL inhibitor; UH15-38, RIPK3 inhibitor; NSA, pyroptosis pathway inhibitor; and *z*-VAD-fmk, apoptosis pathway inhibitor. ns, no significance; *⁣*^*∗*^*p* <  0.05; *⁣*^*∗∗*^*p* <  0.01; *⁣*^*∗∗∗*^*p* <  0.001; and *⁣*^*∗∗∗∗*^*p* <  0.0001.

PCD is an important host defense mechanism during viral infection [8]. Pyroptosis, apoptosis, and necroptosis were three well-established PCD pathways, which could be activated in viral infections [29, 30]. Here, two different types of cell lines commonly used in the PRV-related study, BHK-21 and BV2 cells, were employed in this study to investigate PRV-induced cell death. The results showed that PRV proliferated well in both types of cells (Figure 1A), and the virus could promote cell death in a dose-dependent manner (Figure 1B). To investigate which PCD pathways were involved in PRV-induced cell death, GW806742X (MLKL inhibitor), UH15-38 (RIPK3 inhibitor), NSA (pyroptosis pathway inhibitor), and *z*-VAD-fmk (apoptosis pathway inhibitor) were used to block necroptosis pathway in PRV-infected cells. The results showed that inhibition of RIPK3, MLKL, and apoptosis pathway reduced PRV-induced cell death in both BHK-21 and BV2 cells. However, pyroptosis pathway inhibition had no effect on virus-induced cell death (Figure 1C,D and Supporting Information Figure S1), suggesting PRV can induce cell death through RIPK3- and MLKL-dependent mechanisms.

**Figure 2:**
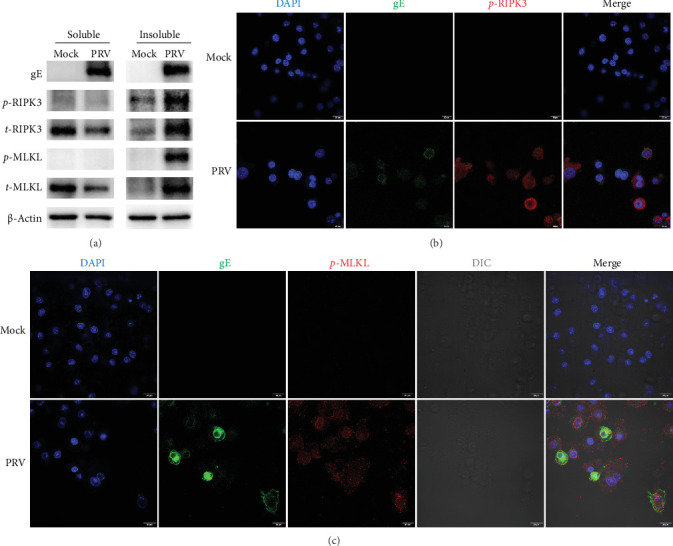
PRV infection induced RIPK3/MLKL-dependent necroptosis in BV2 microglial cells. (A) Activation of RIPK3 and MLKL was examined in both soluble and insoluble protein fractions from PRV-infected BV2 microglial cells (MOI = 5, 24 h postinfection) using western blot analysis. (B and C) Subcellular localization of *p*-RIPK3 and *p*-MLKL in BV2 microglial cells after PRV infection (MOI = 2, 24 h postinfection) by confocal microscopy. Scale bar: 20 μm.

The phosphorylation of RIPK3 (*p*-RIPK3) and MLKL (*p*-MLKL) in cells was considered the hallmarks of necroptosis activation [31]. To investigate PRV-induced necroptosis and phosphorylation of RIPK3 and MLKL during PRV infection, BV2 microglial cells were infected with PRV for 24 h, and *p*-RIPK3/*p*-MLKL expression was analyzed. The results demonstrated that the expression levels of *p*-RIPK3 and *p*-MLKL were significantly upregulated in the insoluble fraction of PRV-infected BV2 microglial cells (Figure 2A). Meanwhile, the confocal microscopy showed that the phosphorylation of RIPK3 and MLKL was significantly activated in BV2 microglial cells infected with PRV (Figure 2B,C). These results confirmed that PRV infection can induce RIPK3- and MLKL-dependent necroptosis in BV2 microglial cells.

### 3.2. ZBP1 Interacted With RIPK3 and Activated RIPK3/MLKL-Dependent Necroptosis During PRV Infection

**Figure 3:**
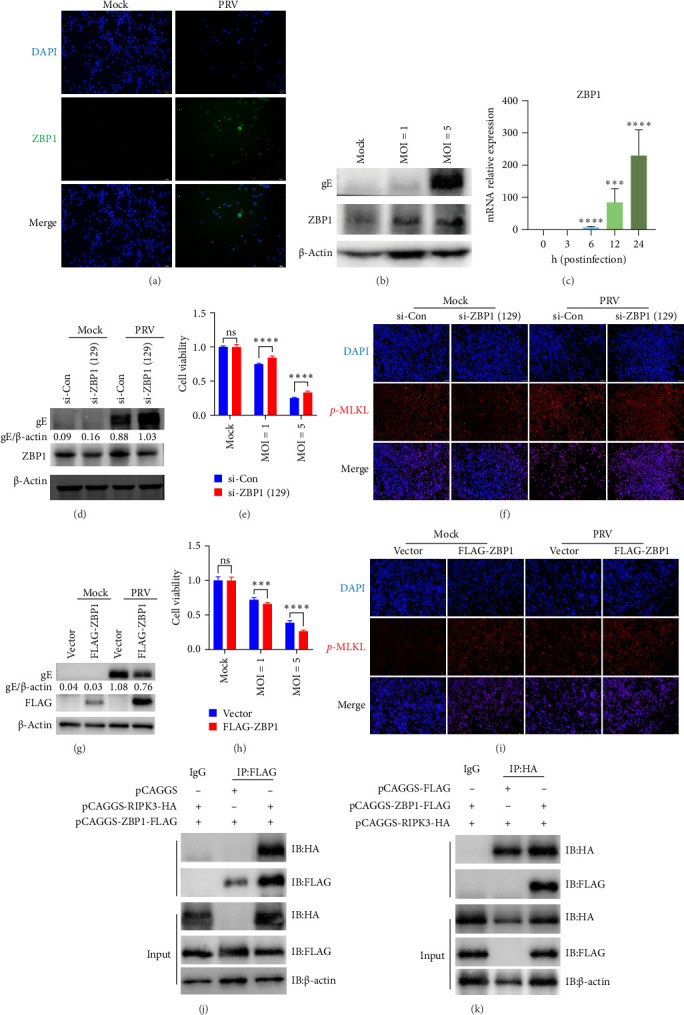
ZBP1 activated RIPK3-dependent necroptosis during PRV infection. (A) The ZBP1 expression in both mock and PRV-infected cells (MOI = 2, 24 h postinfection) was analyzed by IFA (magnification, × 100). (B) Western-blot analysis of ZBP1 in BV2 microglial cells after PRV infection (MOI = 1 or 5, 24 h postinfection). (C) The transcription levels of ZBP1 in PRV-infected BV2 microglial cells (MOI = 5) at the indicated times. (D) Western-blot analysis of PRV gE expression in si-Con- and si-ZBP1-treated BV2 microglial cells. Band intensities were quantified using ImageJ. (E) Cell viability of virus-infected BV2 microglial cells following ZBP1 knockdown. (F) Following ZBP1 knockdown with si-ZBP1 (129) in BV2 microglial cells, *p*-MLKL expression was assessed by IFA in both PRV and mock-infected cells (magnification, × 100). (G and H) Effect of ZBP1 overexpression on PRV infectivity and cell viability of virus-infected BV2 microglial cells. The levels of PRV gE protein were detected by western blot and quantified by Image J. (I) Following ZBP1 overexpression in BV2 microglial cells, *p*-MLKL levels were assessed via IFA in both PRV and mock-infected cells (magnification, × 100). (J and K) ZBP1 was found to interact with RIPK3 in HEK-293T cells via Co-IP assays. ns, no significance; *⁣*^*∗∗∗*^*p* <  0.001; and *⁣*^*∗∗∗∗*^*p* <  0.0001.

ZBP1, a *Z*-nucleic acid-binding protein, has been shown to recruit RIPK3 for necroptosis signaling activation [22]. Additionally, Ma et al. [32] demonstrated that ZBP1 could mediate NLRP3 inflammasome activation during PRV infection, which was then counteracted by PRV-encoded protein VP22. In our study, we found that the expression levels of ZBP1 in BV2 microglial cells were upregulated upon PRV infection in both a viral dose-dependent and time-dependent manner (Figure 3), suggesting that ZBP1 may play a critical role in PRV infection of BV2 microglial cells. To further explore the potential role of ZBP1 in PRV-induced necroptosis, ZBP1 expression was knocked down in BV2 microglial cells using two specific siRNAs. qRT-PCR analysis confirmed efficient knockdown, with si-ZBP1 (129) showing superior knockdown efficiency (Supporting Information Figure S2). Knockdown of ZBP1 using si-ZBP1 (129) in subsequent experiments enhanced PRV replication and cell viability of PRV-infected BV2 microglial cells (Figure 3D,E), while suppressing phosphorylation of MLKL in both Mock and PRV-infected BV2 microglial cells (Figure 3F). By contrast, overexpression of ZBP1 reduced PRV replication and cell viability in PRV-infected BV2 microglial cells (Figure 3G,H), while enhancing phosphorylation of MLKL in both Mock and PRV-infected BV2 microglial cells (Figure 3I). To determine whether ZBP1 interacts with RIPK3, we cotransfected HEK-293T cells with plasmids expressing FLAG-tagged ZBP1 and HA-tagged RIPK3. Co-IP assays were then performed using anti-FLAG or anti-HA antibodies. Specific interaction bands were detected in both assays (Figure 3J,K), suggesting that ZBP1 had an interaction with RIPK3.

### 3.3. ZBP1-Associated *Z*-DNA Accumulated in PRV-Infected BV2 Microglial Cells

**Figure 4:**
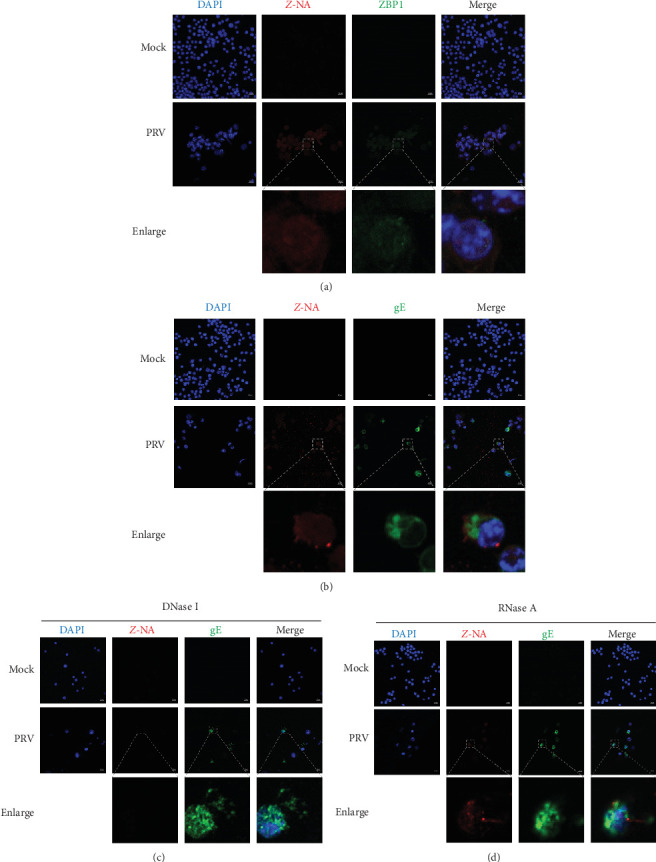
Accumulation of *Z*-DNA was found in PRV-infected BV2 microglial cells. (A) The expression and localization of ZBP1 and *Z*-NA in PRV (MOI = 2)- or mock-infected BV2 microglial cells were observed by staining with antibodies against *Z*-NA and ZBP1. (B–D) PRV- (MOI = 2) or mock-infected BV2 microglial cells were fixed and permeabilized and then either untreated (B), treated with DNase I (1 mg/mL) (C), or RNase A (0.1 mg/mL) (D), and then the cellular localization of *Z*-NA and gE was observed by staining with antibodies against *Z*-NA and gE. Scale bar: 20 μm.

Studies indicated that *Z*-RNA and *Z*-DNA accumulation occurs during IAV and African swine fever virus (ASFV) infections, respectively, which could be sensed by ZBP1 to trigger necroptosis [22, 33]. To investigate whether PRV infection generates *Z*-NA, we performed IFA with an anti-*Z*-NA antibody in PRV-infected BV2 microglial cells. The specific *Z*-NA fluorescence signals were observed in PRV-infected BV2 microglial cells, accompanied by the expression of ZBP1 (Figure 4A). Furthermore, DNase I and RNase A were used to treat PRV-infected cells to distinguish the types of *Z*-NA induced by PRV. The results of IFA showed that DNase I treatment obviously eliminated the *Z*-NA fluorescence signals, while RNase A failed to significantly reduce the *Z*-NA signals in PRV-infected cells (Figure 4).

### 3.4. The Necroptosis Pathway and Its Components RIPK3 and MLKL Independently Restricted PRV Infection in BV2 Microglial Cells

**Figure 5:**
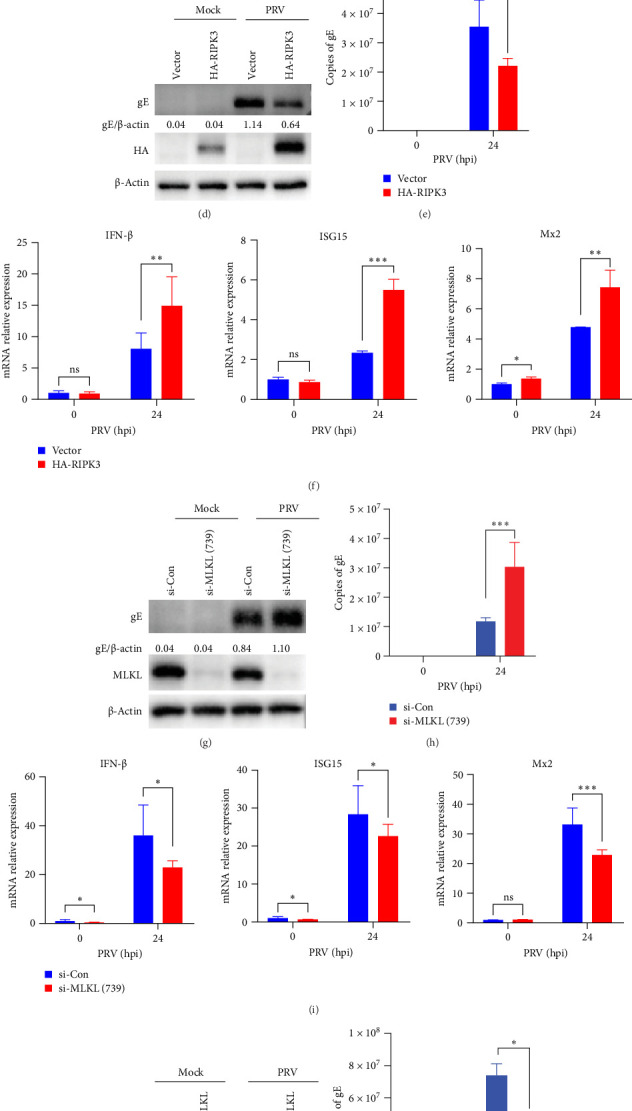
RIPK3 and MLKL restricted PRV infection in BV2 microglial cells by promoting IFN-I activation. (A, B) BV2 microglial cells were transfected with either si-Con or si-RIPK3 (738), followed by mock infection or PRV infection (MOI = 5) for 24 h. PRV gE expression was analyzed by western blot (quantified using ImageJ) and qRT-PCR. (C) The qRT-PCR analysis of IFN-β, ISG15, and Mx2 transcription levels in mock- or PRV (MOI = 5, 24 h postinfection)-infected BV2 microglial cells transfected with si-Con or si-RIPK3 (738). (D and E) BV2 microglial cells were transfected with either empty vector or HA-RIPK3 plasmid, followed by mock infection or PRV infection (MOI = 5) for 24 h. PRV gE expression was analyzed by western blot (quantified using ImageJ) and qRT-PCR. (F) The qRT-PCR analysis of IFN-β, ISG15, and Mx2 transcription levels in mock- or PRV (MOI = 5, 24 h postinfection)-infected BV2 microglial cells transfected with empty vector or HA-RIPK3 plasmid. (G and H) BV2 microglial cells were transfected with either si-Con or si-MLKL (739), followed by mock infection or PRV infection (MOI = 5) for 24 h. PRV gE expression was analyzed by western blot (quantified using ImageJ) and qRT-PCR. (I) The qRT-PCR analysis of IFN-β, ISG15, and Mx2 transcription levels in mock- or PRV (MOI = 5, 24 h postinfection)-infected BV2 microglial cells transfected with si-Con or si-MLKL (739). (J and K) BV2 microglial cells were transfected with either empty vector or HA-MLKL plasmid, followed by mock infection or PRV infection (MOI = 5) for 24 h. PRV gE expression was analyzed by western blot (quantified using ImageJ) and qRT-PCR. (L) The qRT-PCR analysis of IFN-β, ISG15, and Mx2 transcription levels in mock- or PRV (MOI = 5, 24 h postinfection)-infected BV2 microglial cells transfected with empty vector or HA-MLKL plasmid. Band intensities were quantified using ImageJ. ns, no significance; *⁣*^*∗*^*p* <  0.05; *⁣*^*∗∗*^*p* <  0.01; and *⁣*^*∗∗∗*^*p* <  0.001.

To elucidate the functional roles of RIPK3 during PRV infection, we designed two specific siRNAs for RIPK3 knockdown in BV2 microglial cells. Western blot and qRT-PCR revealed that si-RIPK3 (738) achieved the most significant reduction in RIPK3 expression levels (Supporting information Figure S3). Then, RIPK3 knockdown with si-RIPK3 (738) led to a marked increase in PRV replication as evidenced by elevated gE protein expression and transcription levels (Figure 5A,B). Notably, RIPK3 knockdown significantly suppressed the production of IFN-β and downstream ISG expression (ISG15 and Mx2) either in the presence or absence of PRV infection (Figure 5C). Conversely, RIPK3 overexpression significantly reduced PRV infection, as indicated by western blot and qPCR analyses (Figure 5D,E). Notably, PRV infection of RIPK3-overexpressing cells resulted in significantly enhanced transcription of IFN-β and ISGs (ISG15 and Mx2), when compared to control cells (Figure 5F). Similarly, western blot and qRT-PCR results confirmed that the si-MLKL (739) achieved significant MLKL knockdown efficiency in BV2 microglial cells (Supporting Information Figure S4). Silencing of MLKL with si-MLKL (739) significantly increased PRV infection and decreased the levels of IFN-β and ISGs (ISG15 and Mx2) in BV2 microglial cells (Figure 5), whereas overexpression of MLKL significantly reduced PRV infection (Figure 5J,K) and elevated transcription levels of IFN-β and ISGs (ISG15 and Mx2) (Figure 5L). Collectively, these data suggest RIPK3 and MLKL as restriction factors for PRV infection, likely mediated through IFN-β pathway activation.

**Figure 6:**
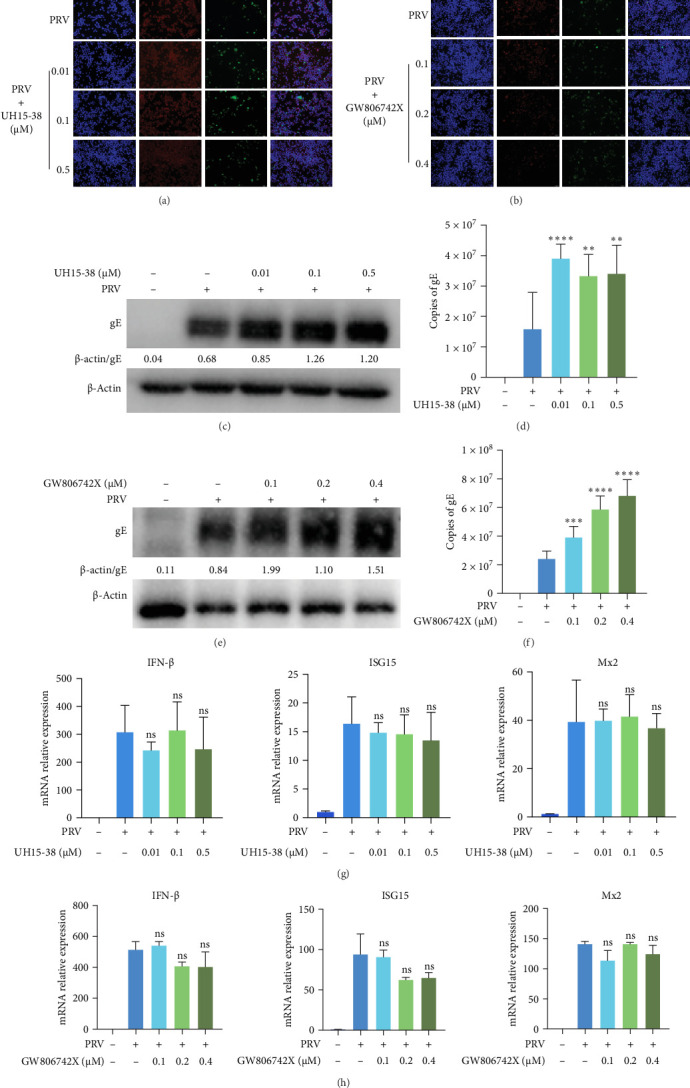
Inhibition of necroptosis pathway promoted PRV infection in BV2 microglial cells. (A) BV2 microglial cells were pretreated with UH15-38 (0.01, 0.1, and 0.5 μM) for 1 h, and then PRV JS-2012 (MOI = 5) was used to infect BV2 microglial cells for 24 h. The expression of PRV gE and *p*-RIPK3 was visualized by IFA. (B) BV2 microglial cells were pretreated with GW806742X (0.1, 0.2, and 0.4 μM) for 1 h, and then PRV JS-2012 (MOI = 5) was used to infect BV2 microglial cells for 24 h. The expression of PRV gE and *p*-MLKL protein was visualized by IFA. (C and D) BV2 microglial cells were pretreated with UH15-38 (0.01, 0.1, and 0.5 μM) for 1 h, and then PRV JS-2012 (MOI = 5) was used to infect BV2 microglial cells for 24 h. Then, PRV gE expression levels were analyzed by western blot (quantified using ImageJ) and qRT-PCR. (E and F) BV2 microglial cells were pretreated with GW806742X (0.1, 0.2, and 0.4 μM) for 1 h, and then PRV JS-2012 (MOI = 5) was used to infect BV2 microglial cells for 24 h. Then, the expression levels of PRV gE were assessed by western blot (quantified using ImageJ) and qRT-PCR. (G and H) BV2 microglial cells were pretreated with UH15-38 or GW806742X for 1 h and then infected with PRV (MOI = 5) for 24 h. The IFN-β, ISG15, and Mx2 mRNA levels were measured by qRT-PCR. ns, no significance; *⁣*^*∗∗*^*p* <  0.01; *⁣*^*∗∗∗*^*p* <  0.001; and *⁣*^*∗∗∗∗*^*p* <  0.0001. Scale bar: 50 μm.

Next, to investigate whether RIPK3/MLKL-mediated necroptosis signaling affects PRV infection, the MLKL inhibitor GW806742X and RIPK3 inhibitor UH15-38 were used to block necroptosis activation during PRV infection, respectively. The results of IFA showed that UH15-38 and GW806742X effectively suppressed PRV-induced phosphorylation of RIPK3 and MLKL, respectively (Figure 6A,B). Meanwhile, both UH15-38 and GW806742X enhanced PRV infection, as indicated by western blot and qRT-PCR (Figure 6). Notably, pharmacological inhibition of RIPK3 or MLKL activation did not affect the transcription levels of IFN-β or downstream ISGs (ISG15 and Mx2) in PRV-infected BV2 microglial cells (Figure 6G,H), indicating that the necroptosis pathway exerts anti-PRV activity through an IFN-I-independent mechanism.

## 4. Discussion

PRV, a neurotropic alphaherpesvirus, naturally infects pigs, leading to severe clinical manifestations, such as reproductive failure, respiratory disease, and fatal encephalitis, resulting in substantial economic losses to the global pig industry [2, 34]. Notably, PRV exhibits broad host tropism, capable of infecting multiple nonnatural hosts, including humans, where it may cause severe neurological symptoms, such as encephalitis [35–37], highlighting its potential zoonotic risk. Within the host central nervous system (CNS), microglia, as the most abundant brain-resident myeloid cells, play a pivotal role in defending against neurotropic pathogen infections [38, 39]. Microglial depletion studies suggest that microglia play a crucial role in inhibiting RNA virus replication and alleviating their lethality in mice models [40–42]. Similarly, a previous study has demonstrated that PRV infection induces microglial activation in both murine and porcine models, with microglia exerting anti-PRV effects during viral early infection [43]. Despite these findings, the precise molecular mechanisms governing PRV–microglia interactions remain poorly understood. Elucidating these mechanisms could provide novel insights into host defense strategies and potential therapeutic interventions against PRV infection.

Necroptosis, a caspase-independent PCD, has been considered a critical antiviral defense mechanism against DNA viruses as well as RNA viruses [17]. The activation of this pathway is characterized by RIPK3/MLKL phosphorylation and necrosome assembly [33]. In this study, pharmacological inhibition of necroptosis revealed that PRV not only triggers necroptosis in BHK-21 cells but also induces it in murine microglia (BV2 microglial cells). Furthermore, the significant expression of *p*-RIPK3 and *p*-MLKL in PRV-infected BV2 microglial cells was demonstrated by western blot and IFA, indicating the potential involvement of necroptosis in the virus–microglia interplay.

ZBP1 has been reported to be capable of mediating the activation of necroptosis induced by various DNA viruses, such as HSV-1 [44], ASFV [33], and porcine parvovirus [26], and RNA viruses, such as IAV [22] and SARS-CoV-2 [45]. A recent study reported that ZBP1 could act as a restrictive factor by triggering the activation of the NLRP3 inflammasome [32]; however, the role of ZBP1 in initiating necroptosis following PRV infection had not been elucidated. In this study, our results indicated that ZBP1 expression is significantly upregulated in PRV-infected BV2 microglial cells in a dose- and time-dependent manner. Ablation of ZBP1 through RNA interference not only enhanced viral replication and host cell viability but also attenuated MLKL phosphorylation. Conversely, ZBP1 overexpression exerted opposing effects, restricting viral propagation while promoting MLKL activation and cell death. Importantly, Co-IP confirmed a physical interaction between ZBP1 and RIPK3, suggesting their functional cooperation in regulating PRV-induced necroptosis. Collectively, these data indicated that ZBP1 served as a critical mediator of PRV infection outcomes and RIPK3/MLKL-mediated necroptosis activation.

Previous studies indicated that the intracellular *Z*-form DNA or RNA could activate ZBP1, which then interacted with RIPK3/MLKL-mediated necroptotic signaling [22, 46]. Here, we demonstrated the formation of ZBP1-associated *Z*-NA structures in PRV-infected BV2 microglial cells by IFA analysis. Furthermore, we performed enzymatic digestion experiments and demonstrated that DNase I, but not RNase A, effectively abolished *Z*-NA fluorescence signals. These results indicated that PRV infection primarily induced *Z*-form DNA, suggesting a *Z*-DNA-dependent mechanism for ZBP1 activation during PRV infection.

During IAV infection, necroptosis could be beneficial to the host when controlled properly. However, this type of cell death could become harmful if triggered in excess or in the wrong cell type or tissue [47]. To investigate the effects of necroptosis on the infection caused by PRV, we adopted the MLKL inhibitor (GW806742X) and RIPK3 inhibitor (UH15-38) to block necroptosis activation during PRV infection, respectively. Intriguingly, blockade of either RIPK3 or MLKL activation enhanced PRV infection in BV2 microglial cells. Notably, unlike previously reported mechanisms [48, 49], inhibition of these necroptotic mediators in this study did not affect IFN-β production, suggesting that the necroptosis pathway may directly restrict PRV infection in BV2 microglial cells via an IFN-independent mechanism. Additionally, we also employed RNA interference and ectopic protein overexpression in BV2 microglial cells to investigate how RIPK3 and MLKL respectively regulate the infection of PRV. Interestingly, knockdown of either RIPK3 or MLKL markedly enhanced PRV replication, while suppressed the production of IFN-β and ISGs; Conversely, either RIPK3 or MLKL overexpression suppressed viral infection, while simultaneously upregulating IFN-β and ISGs. Notably, even in mock-infected cells, knockdown of RIPK3 resulted in decreased expression of IFN-β and ISGs. Previous study demonstrated that lipopolysaccharide (LPS) can activate RIPK3 via TLR4 in macrophages, thereby directly facilitating IFN-β production via the TRIF-IRF3 signaling pathway without inducing MLKL-dependent necroptosis [49]. Consistent with this observation, RIPK3 knockout was found to markedly abrogate the expression of both IFN-β and ISG15 in HT-29 cells [50]. These findings suggest that RIPK3 broadly regulates innate immune responses and modulates basal IFN-β and ISG expression even in uninfected conditions, and meanwhile both RIPK3 and MLKL may intrinsically restrict PRV infection through mechanisms that involve the IFN-I pathway activation, although the precise molecular regulatory mechanisms remain to be elucidated.

**Figure 7:**
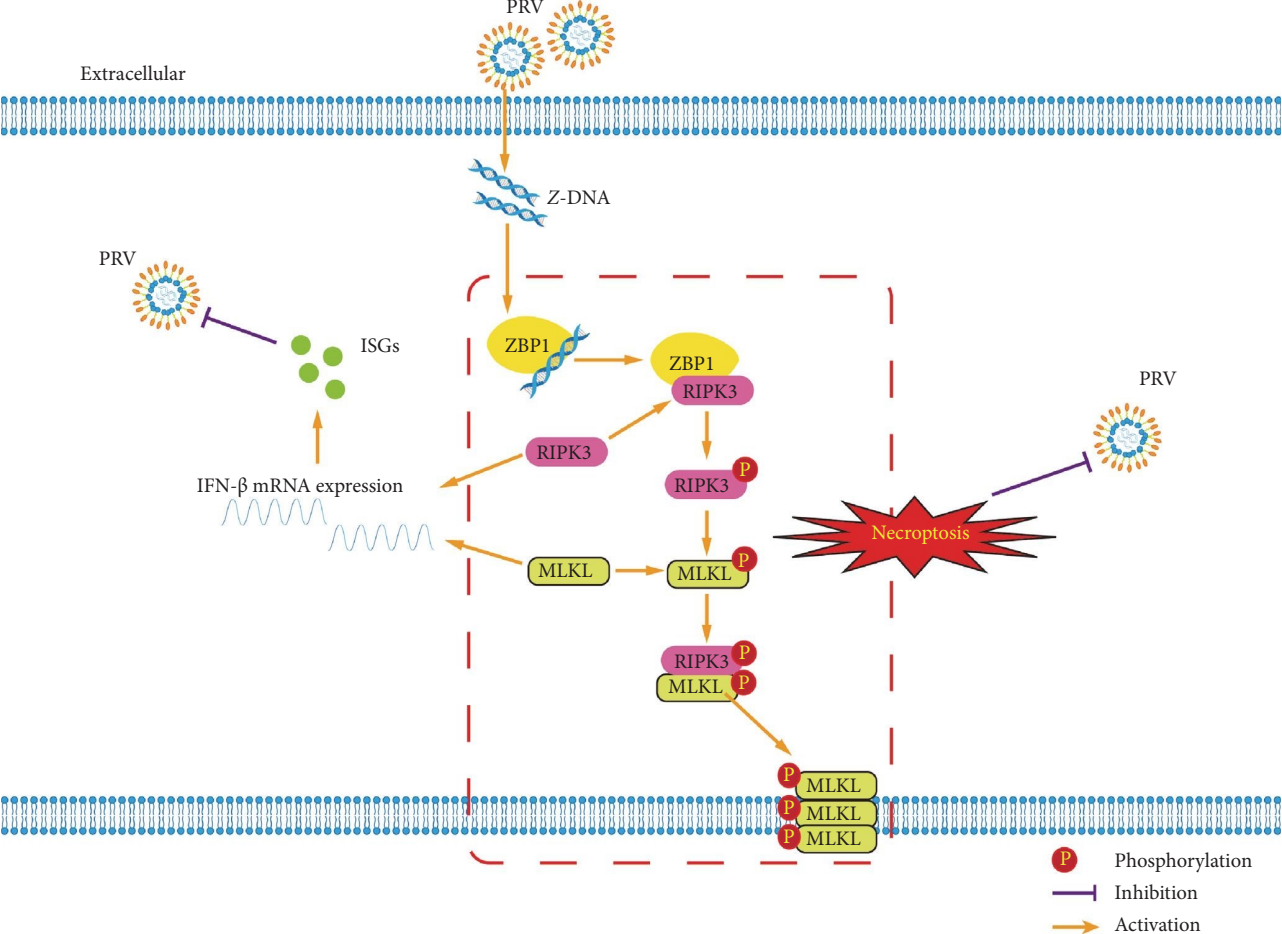
Model of the main mechanism in this study. PRV infection generates *Z*-DNA, which activates ZBP1 and triggers RIPK3/MLKL-dependent necroptosis signaling. During PRV infection, both RIPK3 and MLKL can independently enhance IFN-β signaling activation to inhibit PRV infection. Furthermore, the ZBP1/RIPK3/MLKL necroptosis signaling axis restricts PRV infection in BV2 microglial cells through an IFN-I-independent mechanism.

In conclusion, this study uncovered that PRV infection induced the generation of *Z*-DNA, which was recognized by ZBP1 to induce RIPK3/MLKL-mediated necroptosis (Figure 7). Notably, activation of ZBP1/RIPK3/MLKL-dependent necroptosis by PRV could restrict viral infection in microglia (Figure 7), which provided novel insights into the antiviral mechanisms of microglia in CNS and suggested that necroptosis may act as a therapeutic target in controlling of PRV infection.

## Figures and Tables

**Table 1:** Primer sequences for qRT-PCR.

Primer name	Sequence (5′–3′)
ZBP1-*F*	GACGACAGCCAAAGAAGTGA
ZBP1-*R*	GAGCTATGTCTTGGCCTTCC
RIPK3-*F*	TCTGTCAAGTTATGGCCTACTGG
RIPK3-*R*	GGAACACGACTCCGAACCC
MLKL-*F*	AATTGTACTCTGGGAAATTGCCA
MLKL-*R*	TCTCCAAGATTCCGTCCACAG
IFN-β-*F*	GAGTTACACTGCCTTTGCCATCC
IFN-β-*R*	ACTGTCTGCTGGTGGAGTTCAT
ISG15-*F*	GGTGTCCGTGACTAACTCCAT
ISG15-*R*	CTGTACCACTAGCATCACTGTG
Mx2-*F*	CAAGGAACACCCTCATTTCAGAG
Mx2-*R*	GCAGCTCCTCACTTGCACT
β-Actin-*F*	TGGAATCCTGTGGCATCCATGAAAC
β-Actin-*R*	TAAAACGCAGCTCAGTAACAGTCCG

**Table 2:** Primer sequences for plasmids construction.

Primer name	Sequence (5′–3′)
ZBP1-FLAG-*F*	ATCATTTTGGCAAAGAATTCATGGCAGAAGCTCCTGTTGACTTGA
ZBP1-FLAG-*R*	GCATCGATGAGCTCGAATTCTTGCTTGCTCAGTCCTGTGTCT
RIPK3-HA-*F*	ATGCTCGAGATATGTCTTCTGTCAAGTTATGGCC
RIPK3-HA-*R*	GGAAAAAGATCCGATTCAAGCGTAATCTGGAACATCGTATG GGTACTTGTGGAAGGGCTGCCAG
MLKL-HA-*F*	CGATGCATGCTCGAGATATGGATAAATTGGGACAGATCATCA
MLKL-HA-*R*	GAGGGAAAAAGATCCGATTCAAGCGTAATCTGGAACATCGT ATGGGTACACCTTCTTGTCCGTGGATTC

**Table 3:** Sequences of siRNA.

Name	Sense (5′–3′)
si-Con-*F*	UUCUCCGAACGUGUCACGUTT
si-Con-*R*	ACGUGACACGUUCGGAGAATT
si-ZBP1 (129) -*F*	CCCUCAAUCAAGUCCUUUATT
si-ZBP1 (129) -*R*	UAAAGGACUUGAUUGAGGGTT
si-ZBP1 (411) -*F*	GACAGACGUGGAAGAUCUATT
si-ZBP1 (411) -*R*	UAGAUCUUCCACGUCUGUCTT
si-RIPK3 (738) -*F*	CCCUCUAACAUUCUGCUGGAUTT
si-RIPK3 (738) -*R*	AUCCAGCAGAAUGUUAGAGGGTT
si-RIPK3 (1461) -*F*	CCUCAGAUUCCACAUACUUUATT
si-RIPK3 (1461) -*R*	UAAAGUAUGUGGAAUCUGAGGTT
si-MLKL (739) -*F*	CAUUGGAAUACCGUUUCAGAUTT
si-MLKL (739) -*R*	AUCUGAAACGGUAUUCCAAUGTT
si-MLKL (1426) -*F*	CGGACAGCAAAGAGCACUAAATT
si-MLKL (1426) -*R*	UUUAGUGCUCUUUGCUGUCCGTT

## Data Availability

All data are available within the article and from the corresponding author on reasonable request.

## Supporting Information

Supporting Information Figure S1: Cell viability of BHK-21 and BV2 microglial cells following exposure to various inhibitors. Cells were pretreated with inhibitors for 1 h and then cultured for 24 h. GW806742X, MLKL inhibitor; UH15-38, RIPK3 inhibitor; NSA, pyroptosis pathway inhibitor; and *z*-VAD-fmk, apoptosis pathway inhibitor. ns, no significance; *⁣*^*∗*^*p* < 0.05; *⁣*^*∗∗*^*p* < 0.01; *⁣*^*∗∗∗*^*p* < 0.001; and *⁣*^*∗∗∗∗*^*p* < 0.0001. Figure S2: The qRT-PCR quantification of ZBP1 expression in Mock- or PRV-infected BV2 microglial cells transfected with si-Con, si-ZBP1 (129) or ZBP1 (411). *⁣*^*∗∗*^*p* < 0.01 and *⁣*^*∗∗∗∗*^*p* < 0.0001. Figure S3: Western blot and qRT-PCR analyses of RIPK3 expression in BV2 microglial cells transfected with si-Con, si-RIPK3 (738), or RIPK3 (1461). *⁣*^*∗∗∗∗*^*p* < 0.0001. Figure S4: Western blot and qRT-PCR analyses of MLKL expression in BV2 microglial cells transfected with si-Con, si-MLKL (739), or MLKL (1426). *⁣*^*∗∗∗∗*^*p* < 0.0001.
