# Supplementary material for: Activation of ZBP1/RIPK3/MLKL-Dependent Necroptosis by Pseudorabies Virus Restricts Viral Infection in BV2 Microglia Cells
Source: Transbound Emerg Dis. 2025 Oct 7;2025:8510846. doi: 10.1155/tbed/8510846 (PMC12520810; doi:10.1155/tbed/8510846)

**Supporting Information**

Figure S1. Cell viability of BHK-21 and BV2 microglial cells following exposure to various inhibitors. Cells were pretreated with inhibitors for 1 h and then cultured for 24 h. GW806742X: MLKL inhibitor; UH15-38: RIPK3 inhibitor; NSA: pyroptosis pathway inhibitor; and z-VAD-fmk: apoptosis pathway inhibitor. ns, no significance; **p* < 0.05, ***p* < 0.01, ****p* < 0.001 and *****p* < 0.0001.

Figure S1.


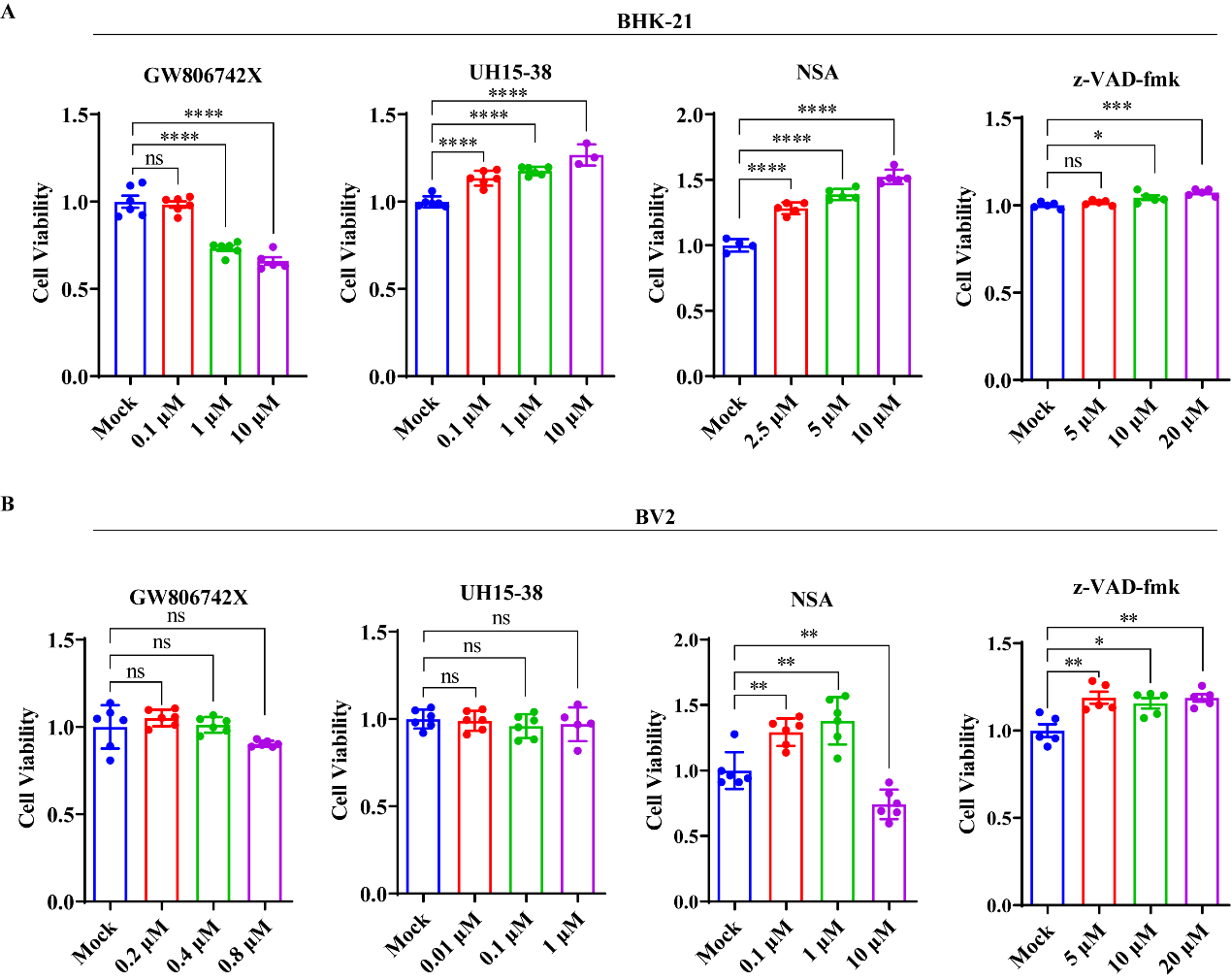


Figure S2. The qRT-PCR quantification of ZBP1 expression in Mock- or PRV-infected BV2 microglial cells transfected with si-Con, si-ZBP1 (129) or ZBP1 (411). ***p* < 0.01 and *****p* < 0.0001.

Figure S2.


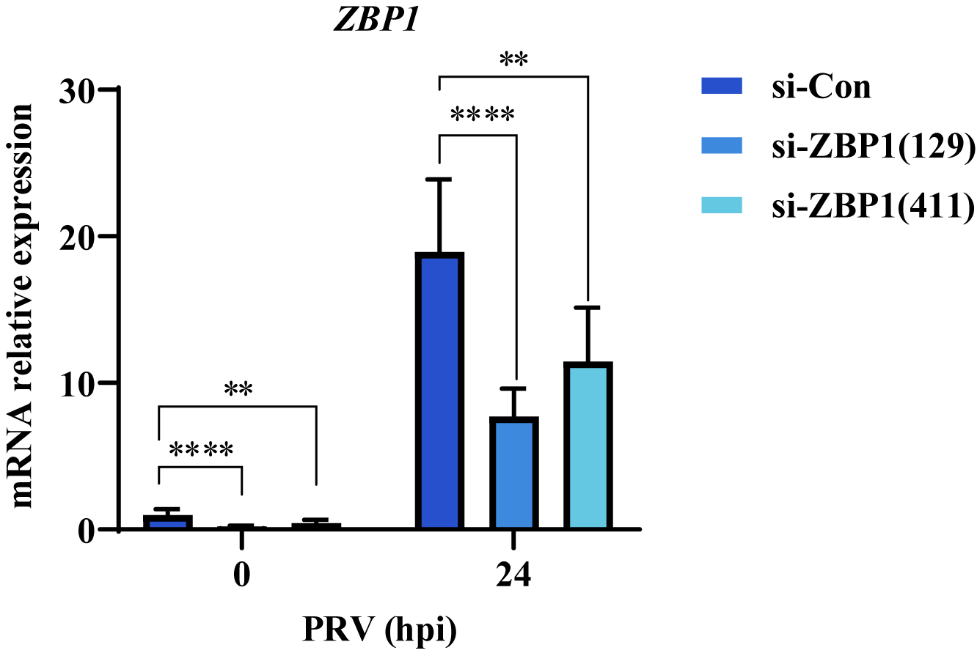


Figure S3. Western blot and qRT-PCR analyses of RIPK3 expression in BV2 microglial cells transfected with si-Con, si-RIPK3 (738) or RIPK3 (1461). *****p* < 0.0001.

Figure S3.


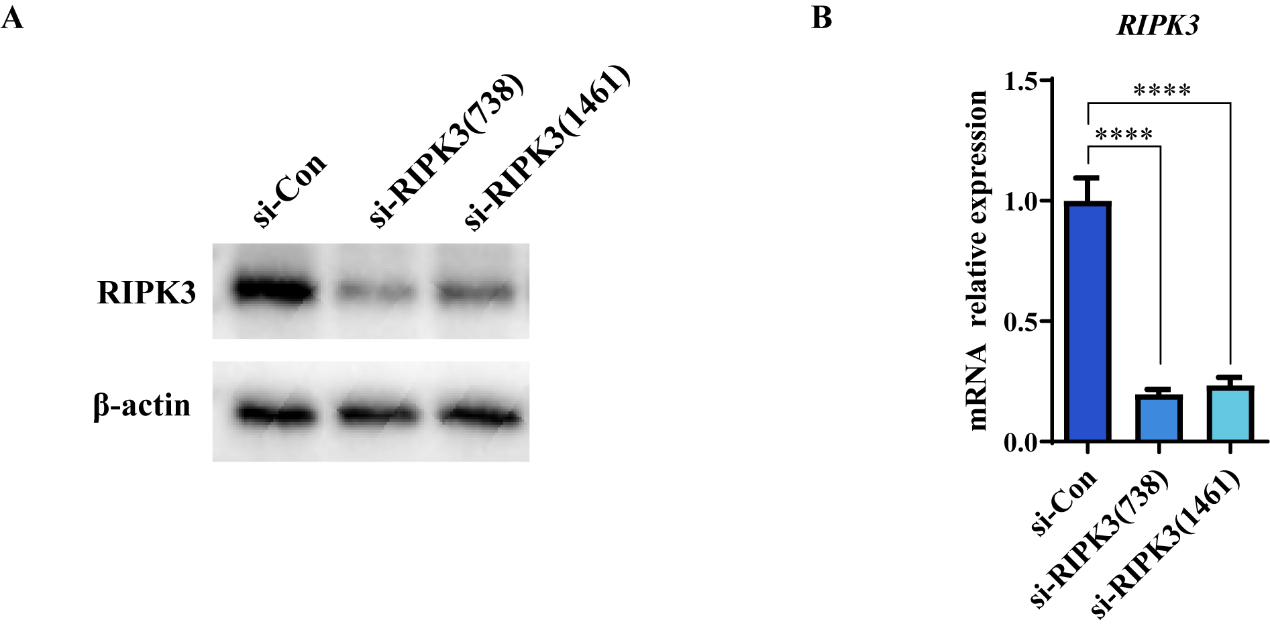


Figure S4. Western blot and qRT-PCR analyses of MLKL expression in BV2 microglial cells transfected with si-Con, si-MLKL (739) or MLKL (1426). ****p < 0.0001.

Figure S4.


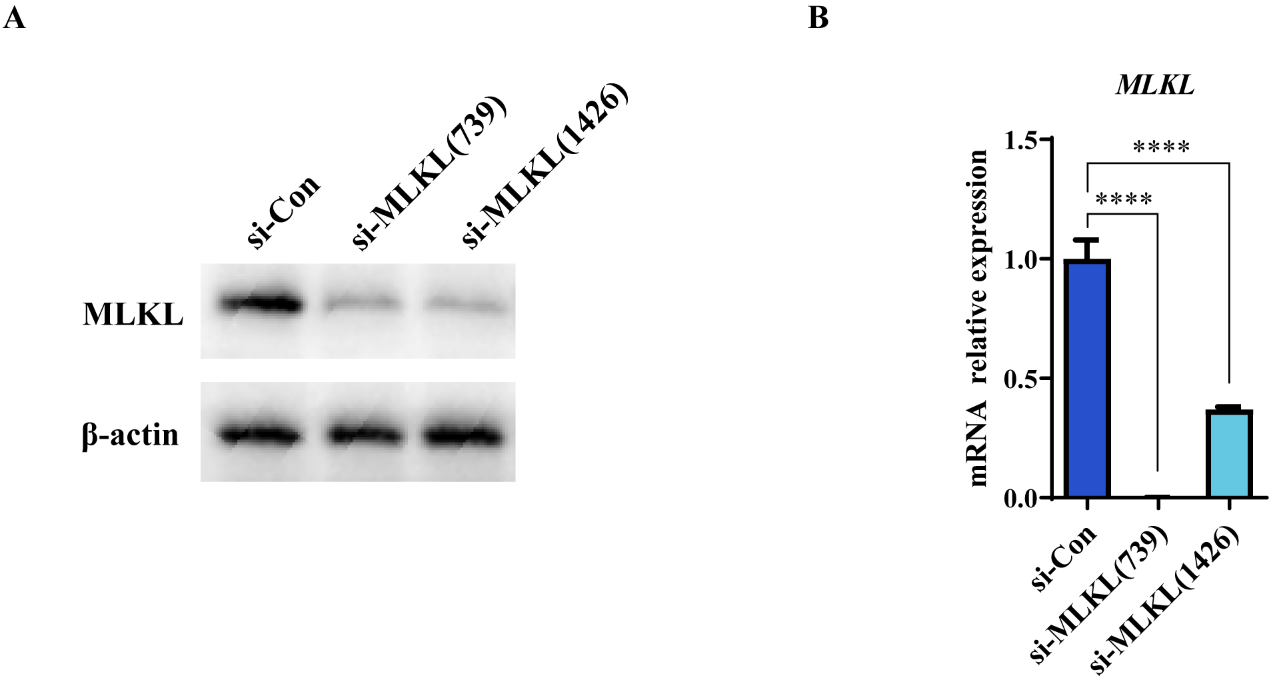

Supplement: Supporting Information — Figure S1: Cell viability of BHK-21 and BV2 microglial cells following exposure to various inhibitors. Cells were pretreated with inhibitors for 1 h and then cultured for 24 h. GW806742X, MLKL inhibitor; UH15-38, RIPK3 inhibitor; NSA, pyroptosis pathway inhibitor; and z-VAD-fmk, apoptosis pathway inhibitor. ns, no significance; ⁣∗p < 0.05; ⁣∗∗p < 0.01; ⁣∗∗∗p < 0.001; and ⁣∗∗∗∗p < 0.0001. Figure S2: The qRT-PCR quantification of ZBP1 expression in Mock- or PRV-infected BV2 microglial cells transfected with si-Con, si-ZBP1 (129) or ZBP1 (411). ⁣∗∗p < 0.01 and ⁣∗∗∗∗p < 0.0001. Figure S3: Western blot and qRT-PCR analyses of RIPK3 expression in BV2 microglial cells transfected with si-Con, si-RIPK3 (738), or RIPK3 (1461). ⁣∗∗∗∗p < 0.0001. Figure S4: Western blot and qRT-PCR analyses of MLKL expression in BV2 microglial cells transfected with si-Con, si-MLKL (739), or MLKL (1426). ⁣∗∗∗∗p < 0.0001. [file 8510846.f1.docx]
